# Antiplatelet treatment in acute coronary syndrome patients: Real-world data from the START-Antiplatelet Italian Registry

**DOI:** 10.1371/journal.pone.0219676

**Published:** 2019-07-15

**Authors:** Rossella Marcucci, Giuseppe Patti, Paolo Calabrò, Anna Maria Gori, Guido Grossi, Plinio Cirillo, Vittorio Pengo, Paolo Gresele, Pasquale Pignatelli, Emilia Antonucci, Carlo di Mario, Serafina Valente, Gualtiero Palareti

**Affiliations:** 1 Department of Experimental and Clinical Medicine, University of Florence, Florence, Italy; 2 Campus Bio‐Medico University of Rome, Rome, Italy; 3 Division of Cardiology, Monaldi Hospital and "Luigi Vanvitelli" University of Campania, Naples, Italy; 4 Department of Advanced Biomedical Sciences, School of Medicine, "Federico II" University, Naples, Italy; 5 Department of Cardiothoracic and Vascular Sciences, University Hospital of Padua, Padua, Italy; 6 Department of Medicine, Division of Internal and Cardiovascular Medicine, University of Perugia, Perugia, Italy; 7 Department of Internal Medicine and Medical Specialties, La Sapienza University of Rome, Rome, Italy; 8 Arianna Anticoagulazione Foundation, Bologna, Italy; 9 Structural Interventional Cardiology, Cardio-toraco-vascular Department, Careggi University Hospital, Florence, Italy; University of Bologna, ITALY

## Abstract

**Background:**

Despite great advances with the introduction of ticagrelor and prasugrel in the treatment of acute coronary syndromes (ACS), the risk of thrombosis and bleeding remains significant and affects the choice of clinicians in the treatment of the single patient. Large registries are effective tools to explore patterns of drug administration and adherence to guideline recommendations in real-world clinical practice.

**Methods:**

START- antiplatelet is a prospective, observational registry carried out by seven Italian cardiology institutions on patients admitted for ACS aimed to document the real world treatment of ACS patients, adding also data on 12-month follow-up. We present data on the first 1050 patients who have completed 1-year follow-up on a total of 1537 patients. Primary end-points were: 1) MACCE (Major Adverse Cardiovascular and Cerebrovascular Events) including all-cause and cardiovascular mortality, non fatal MI, urgent revascularization, TIA and ischemic stroke; 2) Major and minor bleeding according to TIMI, GUSTO and ISTH classifications

**Results:**

The dual antiplatelet treatment most prescribed was aspirin plus ticagrelor (47.9%) and aspirin plus clopidogrel (32.1%). At a mean follow-up was 335±131 days, both ticagrelor and prasugrel are associated with a statistically significant reduced total and cardiovascular mortality. Both prasugrel and ticagrelor do not show a significant increased incidence of major and minor bleedings with respect to clopidogrel. Patients with monotherapy had significantly higher incidence of both ischemic stroke and major bleedings.

**Discussion:**

The analysis of the register has documented that both ticagrelor and prasugrel are associated with a statistically significant reduced total and cardiovascular mortality but both do not show a significant increased incidence of major and minor bleedings with respect to clopidogrel.

## Introduction

Antiplatelet treatment is a cornerstone of the management of patients with acute coronary syndrome (ACS).

In recent years several new antiplatelet drugs have been introduced and the most recent guidelines suggest the use of the new potent antiplatelets–prasugrel and ticagrelor–in addition to aspirin after an ACS [1–2]. Despite great advances with these therapies, the risk of thrombosis and bleeding remains significant and affects the choice of clinicians in the treatment of the single patient. Large registries are effective tools to explore patterns of drug administration and adherence to guideline recommendations in real-world clinical practice.

START- antiplatelet is a prospective, observational registry carried out by seven Italian cardiology institutions on patients admitted for ACS aimed to document the real world treatment of ACS patients, adding also data on 12-month follow-up [3].

## Material and methods

One thousand five hundreds and thirty- seven patients were enrolled in the Register at May 31, 2017. In this paper we present data on the first 1050 patients who have completed 1-year follow-up on a total of 1537 patients. Inclusion criteria were: age ≥18 years; written informed consent for study participation; admission for ACS (either STEMI or NSTE-ACS). To reduce selection bias, no explicit exclusion criteria were present; moreover, two specific and fixed working days in the week (for example Tuesday and Friday) were chosen at each site and all consecutive patients with ACS admitted in those days were enrolled.

The study design consisted of a clinical evaluation at the time of hospital discharge (baseline visit), at six-month and at 1-year follow-up. Adherence to antiplatelet treatment was assessed during scheduled or unscheduled examinations.

All other possible information derived from hospital readmission or by the referring physician, relatives, or municipality live registries were entered into the prospective database.

Demographic data, clinical characteristics, risk factors and treatment modalities were collected at baseline; occurrence of adverse events, both cardiovascular events and bleeding complications, was recorded at the 1-year follow-up evaluation, as well as the type of therapy given during follow-up, drug-related side effects, duration and compliance to antithrombotic treatments. Only documented adverse events were considered relevant, as defined in current guidelines and with the date of any event recorded after the baseline visit. Individual data were entered into an electronic case report form including various plausibility checks for the considered variables. The registry was investigators-driven, non-sponsored and was approved by the Ethic Committee of each participating institution.

START-ANTIPLATELET is a branch of the START registry (Survey on anTicoagulated pAtients RegisTer, NCT02219984), promoted by the Arianna Anticoagulazione Foundation, Bologna. The registry was investigators-driven, non-sponsored and was approved by the Ethic Committee of each participating institution (Campus Bio-Medico University of Rome; Monaldi Hospital and "Luigi Vanvitelli" University of Campania; "Federico II" University of Naples; University of Perugia; University Hospital of Padua; La Sapienza University of Rome; University of Florence).

### Clinical end-points

Primary end-points were:

1. MACCE (Major Adverse Cardiovascular and Cerebrovascular Events) including all-cause and cardiovascular mortality, non fatal MI, urgent revascularization, TIA and ischemic stroke. A diagnosis of non-STEMI (NSTEMI) or STEMI was made if ischemic symptoms and one or more of the following were reported: cardiac biomarkers greater than the local laboratory reference range, new ST-T wave changes, new left bundle branch block, pathologic Q waves on the electrocardiogram, or new imaging evidence of loss of viable myocardium or regional wall motion abnormality. Stroke was defined as loss of neurological function caused by an ischemic or hemorrhagic event with residual symptoms 24 hours after onset or leading to death. Unplanned coronary revascularization included any unplanned PCI or coronary artery bypass graft surgery occurring after the index PCI. Staged revascularization procedures planned at the time of index PCI and occurring within 60 days were not considered unplanned revascularization events unless a documented recurrent ischemic episode determined the timing of the follow-up procedure.
2. Major and minor bleeding according to TIMI [4] Global Use of Strategies to Open Occluded Coronary Arteries (GUSTO) [5] and ISTH classifications [6].

TIMI major bleeding was defined as bleeding with a hemoglobin drop of ≥5 g/dL, intracranial hemorrhage or fatal bleeding regardless of whether or not a bleeding site had been identified, while TIMI minor was defined as bleeding with a hemoglobin drop of ≥3 to < 5 g/dL with an identifiedbleeding site or if the patient had spontaneous gross hematuria, hemoptysis, or hematemesis. Events were classified as "loss, no site" if the patient had a decrease in hemoglobin of more than 4 g/dL but not exceeding 5 g/dl without an identified bleeding site [4].

Bleeding events during or subsequent to the index hospitalization were stratified according to the GUSTO criteria into the following categories: mild (not meeting criteria for moderate or severe bleeding), moderate (bleeding requiring blood transfusion but not causing hemodynamic compromise), or severe (intracranial hemorrhage or bleeding causing hemodynamic compromise) [5]. The GUSTO bleeding events were independently validated by study physicians via medical record review.

Criteria of the International Society on Thrombosis and Haemostasis [6], defined major bleeding as fatal bleeding or symptomatic bleeding in critical area or organ, or bleeding causing a fall of hemoglobin level of 2 g/dL (1.24 mmol/L) or more, or leading to transfusion of ≥ 2 units of whole blood or red cells.

### Statistical analysis

Discrete data are expressed as frequencies, and continuous data as mean ± SD or median and interquartile range, as appropriate. The χ^2^ test was used to compare categorical variables, and the unpaired two-tailed Student’s t-test or Mann-Whitney rank-sum test was used to test differences between continuous variables. Survival curves were generated with the use of the Kaplan-Meier method, and the difference between groups was assessed by log-rank test. Multivariable regression analysis to evaluate the independent contribution of clinical, angiographic, procedural, and therapies to the endpoints was performed by the Cox proportional hazards model. Variables found not to be associated with the outcome at univariate analysis with a p value >0.100 were removed from the final, most rigorous, regression model. Variables were: age, gender, BMI≥ 30 Kg/m^2^, LVEF<40%, hypertension, hypercholesterolemia, smoking habit, diabetes, family history of CAD, chronic kidney disease (CKD), previous AMI, previous bleeding, previous PCI, atrial fibrillation (both non valvular and valvular), anticoagulant therapy, antiplatelets drugs, statins, beta-blockers, nitrates, diuretics and duration of dual antiplatelet therapy. Competing risk regression models were used to calculate subdistribution hazard ratios with 95% CI (SHR). These models take into account the fact that individuals who died are no longer at risk for major bleedings or cardiovascular death (i.e. these models correct for the competing risk of death).

The proportional hazard assumption was assessed and satisfied graphically by plotting log (-log) survival curves against log survival time for each predictor category and verifying whether the curves were parallel and, in addition, using time-dependent covariates. A p-value <0.05 was considered significant. All tests were two-sided. Analyses were performed with SPSS statistical package, Version 25 (IBM Corp., Armonk, NY, USA) and Stata statistical package, Version 13 for Mac (Stata Corp, TX, USA).

## Results

Out of a total population of 1537 ACS patients, 1050 patients (68.3%) completed a 12-month follow-up.

Demographic and clinical characteristics of ACS patients who completed a follow-up according to the antiplatelet treatment at the admission were shown in Table 1.

**Table 1:** Demographic and clinical characteristics.

	Monotherapy (N = 68)	Aspirin+Clopidogrel (N = 337)	Aspirin+Prasugrel (N = 142)	Aspirin+Ticagrelor (N = 503)	P-Value
**Age (years), mean (SD)**	73.3 (11.6)	71.9 (12.9)	61.2 (10.5)	65.5 (12.1)	<0.001
**Age ≥ 75 y, n (%)**	38 (55.9)	166 (49.3)	15 (10.6)	126 (25)	<0.001
**Gender, males, n (%)**	39 (57.4)	236 (70)	114 (80.3)	374 (74.4)	0.003
**BMI (Kg/m^2^), mean (SD)**	26.3 (4.4)	26.8 (4.2)	27.5 (4.2)	27.0 (4.1)	0.222
**BMI ≥ 30 Kg/m^2^, n (%)**	14 (20.6)	62 (18.4)	32 (22.5)	102 (20.3)	0.765
**Creatinine (mg/dL), median (IQR)**	1.1 (0.8–1.3)	1.3 (0.8–1.2)	1.0 (0.8–1.1)	1.0 (0.8–1)	<0.001
**Creatinine Clerance (mL/min), mean (SD)**	68.9 (35.3)	72.3 (37.4)	95.7 (24.4)	89.7 (33.4)	<0.001
**Creatinine Clearance:**					
**< 30 mL/mL**	5 (7.4)	37 (11.0)	1 (0.7)	12 (2.4)	<0.001
**30–59 mL/min**	25 (36.8)	99 (29.4)	21 (14.8)	83 (16.5)	
**60–89 mL/min**	22 (32.4)	104 (30.9)	42 (29.6)	167 (33.2)	
**≥ 90 mL/min**	16 (23.5)	97 (28.8)	78 (54.9)	241 (47.9)	
**Haemoglobin (g/dL), mean (SD)**	13.2 (1.8)	13.1 (2.1)	14.2 (1.5)	13.9 (1.8)	<0.001
**LVEF, %, mean (SD)**	53.1 (23.7)	49.6 (14.0)	48.5 (11.8)	47.9 (9.2)	0.010
**LVEF<40%, n (%)**	13 (19.1)	62 (18.4)	15 (10.6)	70 (13.9)	0.090
**Hypertension, n (%)**	56 (82.4)	247 (73.3)	101 (71.1)	357 (71)	0.247
**Hypercholesterolemia, n (%)**	37 (54.4)	184 (54.6)	75 (52.8)	253 (50.3)	0.646
**Smoking habit, n (%)**	27 (39.7)	121 (35.9)	83 (58.5)	287 (57.1)	<0.001
**Diabetes, n (%)**	17 (25.0)	103 (30.6)	38 (26.8)	121 (24.1)	0.215
**Family history for CAD, n (%)**	17 (25.0)	79 (23.4)	48 (33.8)	165 (32.8)	0.015
**Medical history**					
**Myocardial infarction, n (%)**	11 (16.2)	81 (24)	22 (15.5)	85 (16.9)	0.037
**PCI, n (%)**	14 (20.6)	88 (26.1)	18 (12.7)	85 (16.9)	0.001
**Non Valvular Atrial fibrillation**	17 (25.0)	61 (18.1)	4 (2.8)	6 (1.2)	0.001
**Valvular Atrial Fibrillation**	3 (4.4)	9 (2.7)	0 (0)	0 (0)	0.001
**Peripheral Artery Disease**	9 (13.2)	32 (9.5)	4 (2.8)	29 (5.8)	0.007
**Chronic Kidney Disease, n (%)**	5 (7.4)	37 (11.0)	1 (0.7)	13 (2.6)	<0.001
**Cerebrovascular disease**	2 (2.9)	23 (6.8)	1 (0.7)	7 (1.4)	<0.001
**Major Bleeding, n (%)**	4 (5.9)	11 (3.3)	0	5 (1)	0.003
**Minor Bleeding, n (%)**	1 (1.5)	1 (0.3)	0	3 (0.6)	0.479
**Admission**					
**Unstable Angina, n (%)**	29 (42.6)	66 (19.6)	3 (2.1)	33 (6.6)	<0.001
**NSTEMI, n (%)**	26 (38.2)	127 (37.7)	25 (17.6)	192 (38.2)	
**STEMI, n (%)**	13 (19.1)	144 (42.7)	114 (80.3)	278 (55.3)	
**Reperfusion Strategies**					
**CABG, n (%)**	9 (13.2)	9 (2.7)	1 (0.7)	5 (1)	<0.001
**PCI, n (%))**	20 (29.4)	267 (79.2)	136 (95.8)	465 (92.4)	<0.001
**Medical Therapy**	40 (58.8)	66 (19.6)	13 (9.2)	33 (6.6)	<0.001

CABG = coronary artery bypass grafting; PCI = percutaneous coronary intervention; STEMI = ST-segment elevation myocardial infarction

As expected, in the ACS population, male prevalence was high (763/1050, 72.7%) as well as the prevalence of traditional cardiovascular risk factors.

Female ACS patients (n = 287) were significantly older (≥75 years) (47.4% vs 27.4%, p<0.001) and had a higher prevalence of hypertension (77.4% vs 70.6%) and diabetes (30.3% vs 25.2%) than male patients. Smoking habit was more prevalent in male than in female patients (53.7% vs 37.6%).

According to the antiplatelet treatment prescribed at the admission, ACS patients treated with monotherapy (aspirin or clopidogrel) were significantly older than patients treated with aspirin+prasugrel or aspirin+ticagrelor (Table 1).

ACS patients treated with monotherapy were more frequently (42.6%) female than ACS patients treated with a dual antiplatelet therapy (aspirin+clopidogrel, aspirin+prasugrel or aspirin+ticagrelor) (25.1%). The prevalence of smoking habit was higher in ACS patients treated with aspirin+prasugrel or aspirin+ticagrelor than in patients with aspirin+clopidogrel or monotherapy (Table 1).

The ACS patients with monotherapy or aspirin+clopidogrel had more frequently a previous PCI, non valvular atrial fibrillation (NVAF), valvular atrial fibrillation, peripheral arterial disease, and chronic kidney disease with respect to patients treated with aspirin+prasugrel or aspirin+ticagrelor.

### Medical theraphy at discharge

At discharge, the medical therapy was recorded. As expected, the majority of the ACS patients were discharged with statin and PPI therapy (96.5% and 97.4% respectively). Diuretics and nitrates were more frequently prescribed in ACS patients with monotherapy than in ACS patients with dual antiplatelet treatment. Conversely, statins were less frequently prescribed in ACS patients with monotherapy than in ACS patients with dual antiplatelet treatment (Table 2).

**Table 2:** Treatment at discharge.

	Monotherapy (N = 68)	Aspirin+Clopidogrel (N = 337)	Aspirin+Prasugrel (N = 142)	Aspirin+Ticagrelor (N = 503)	P-Value
**Proton Pump Inhibitors, n(%)**	65 (95.6)	323 (95.8)	142 (100)	493 (98.0)	0.033
**Omeprazole**	15 (22.1)	126 (37.4)	74 (52.1)	256 (50.9)	<0.001
**Pantoprazole**	39 (57.4)	135 (40.1)	38 (26.8)	169 (33.6)	
**Lansoprazole**	8 (11.8)	53 (15.7)	27 (19.0)	50 (9.9)	
**Esomeprazole**	2 (2.9)	4 (1.2)	0 (0)	15 (3.0)	
**Rabeprazole**	0 (0)	4 (1.2)	3 (2.1)	3 (0.6)	
**Beta-blockers, n (%)**	52 (76.5)	216 (64.1)	107 (75.4)	352 (70)	0.037
**ACE inhibitors/ARBS, n (%)**	41 (60.3)	206 (61.1)	96 (67.6)	336 (66.8)	0.266
**Diuretics, n (%)**	34 (50)	120 (35.6)	33 (23.2)	108 (21.5)	<0.001
**Nitrates, n (%)**	15 (22.1)	46 (13.6)	11 (7.7)	33 (6.6)	<0.001
**Omega-3 Fatty Acids, n (%)**	0 (0)	3 (0.9)	7 (4.9)	10 (2)	0.017
**Statins, n (%)**	60 (88.2)	319 (94.7)	142 (100)	493 (98)	<0.001
**Atorvastatin, n (%)**	56 (82.4)	305 (90.5)	138 (97.2)	478 (95)	<0.001
**Pravastatin, n (%)**	0 (0)	0 (0)	1 (0.7)	1 (0.2)	
**Rosuvastatin, n (%)**	2 (2.9)	6 (1.8)	2 (1.4)	10 (2)	
**Simvastatin, n (%)**	2 (2.9)	8 (2.4)	1(0.7)	4 (0.8)	
**Vitamin K Antagonists, n(%)**	14 (20.6)	61 (18.1)	3 (2.1)	5 (0)	<0.001
**Direct Oral Anticoagulants, n(%)**	4 (5.8)	15 (4.5)	0 (0)	0 (0)	<0.001

As concerns antiplatelet treatment, at discharge physicians prescribed in less than 8% ACS patients a monotherapy (aspirin or clopidogrel). The dual antiplatelet treatment most prescribed was aspirin plus ticagrelor (45.4%) and aspirin plus clopidogrel (32.5%) (Table 2).

### Clinical outcomes

The mean follow-up was 335±131 days. Table 3 summarizes the clinical outcomes during 1-year follow-up.

**Table 3:** Clinical outcomes at 1-year follow-up.

	Patients (n = 1050)
**Total Mortality, n (%)**	71 (6.8)
**Cardiovascular Mortality, n (%)**	42 (4.0)
**Target Lesion Revascularization, n (%)**	20 (1.9)
**Re-infarction, n (%)**	32 (3.0)
**TIA, n (%)**	1 (0.09)
**Ischemic Stroke n (%)**	9 (0.9)
**Peripheral Embolism, n (%)**	2 (0.2)
**Hemorrhagic Complications**	
**Major Bleeding**	19 (1.8)
**TIMI Major, n (%)**	15 (1.4)
**TIMI Minor, n (%)**	13 (1.2)
**TIMI Minimal, n (%)**	24 (2.3)
**GUSTO Severe or Life-threatening, n (%)**	12 (1.1)
**GUSTO Moderate n (%)**	10 (0.9)
**GUSTO Mild, n (%)**	30 (2.8)
**ISTH Cerebral, n (%)**	8 (0.7)
**ISTH Gastrointestinal, n (%)**	7 (0.6)
**ISTH Loss of Hgb ≥ 2 g/dL, n (%)**	1 (0.09)
**ISTH Pericardial, n (%)**	1 (0.09)
**ISTH Retroperitoneal, n (%)**	1 (0.09)
**Minor Bleeding**	71 (6.8)

As shown in Table 3, at follow-up about 7% of patients died, 4.0% for cardiovascular causes. The incidence of re-infarction in this subgroup of patients was 3.0% (32/1050).

According to antiplatelet therapies prescribed at admission, the incidence rates of all-cause mortality and cardiovascular mortality were significantly (p<0.001) higher in ACS patients treated with a monotherapy (aspirin or clopidogrel) or aspirin plus clopidogrel with respect to patients treated with the combination of aspirin plus prasugrel or aspirin plus ticagrelor (Table 4). Patients with mono-therapy had significantly higher incidence of ischemic events and major bleedings (Table 4).

**Table 4:** Incidence rate of clinical outcomes according to antiplatelet treatment at discharge.

	Monotherapy (N = 68)	Aspirin+Clopidogrel (N = 337)	Aspirin+Prasugrel (N = 142)	Aspirin+Ticagrelor (N = 503)	P-Value
**Total Mortality,** **p-years (95%CI)**	0.210 (0.12–0.37)	0.120 (0.08–0.17)	0.037 (0.016–0.17)	0.045 (0.03–0.07)	0.002
**Cardiovascular Mortality,** **p-years (95%CI)**	0.083 (0.056–0.12)	0.053 (0.017–0.16)	0.030 (0.011–0.08)	0.025 (0.014–0.045)	0.003
**Ischaemic events,** **p-years (95%CI)**	0.127 (0.06–0.27)	0.071 (0.046–0.11)	0.053 (0.025–0.11)	0.066 (0.046–0.095)	0.001
**Haemorrhagic events,** **p-years (95%CI)**	0.530 (0.017–0.16)	0.024 (0.012–0.51)	0.015 (0.004–0.06)	0.015 (0.008–0.033)	0.050

**Fig 1:**
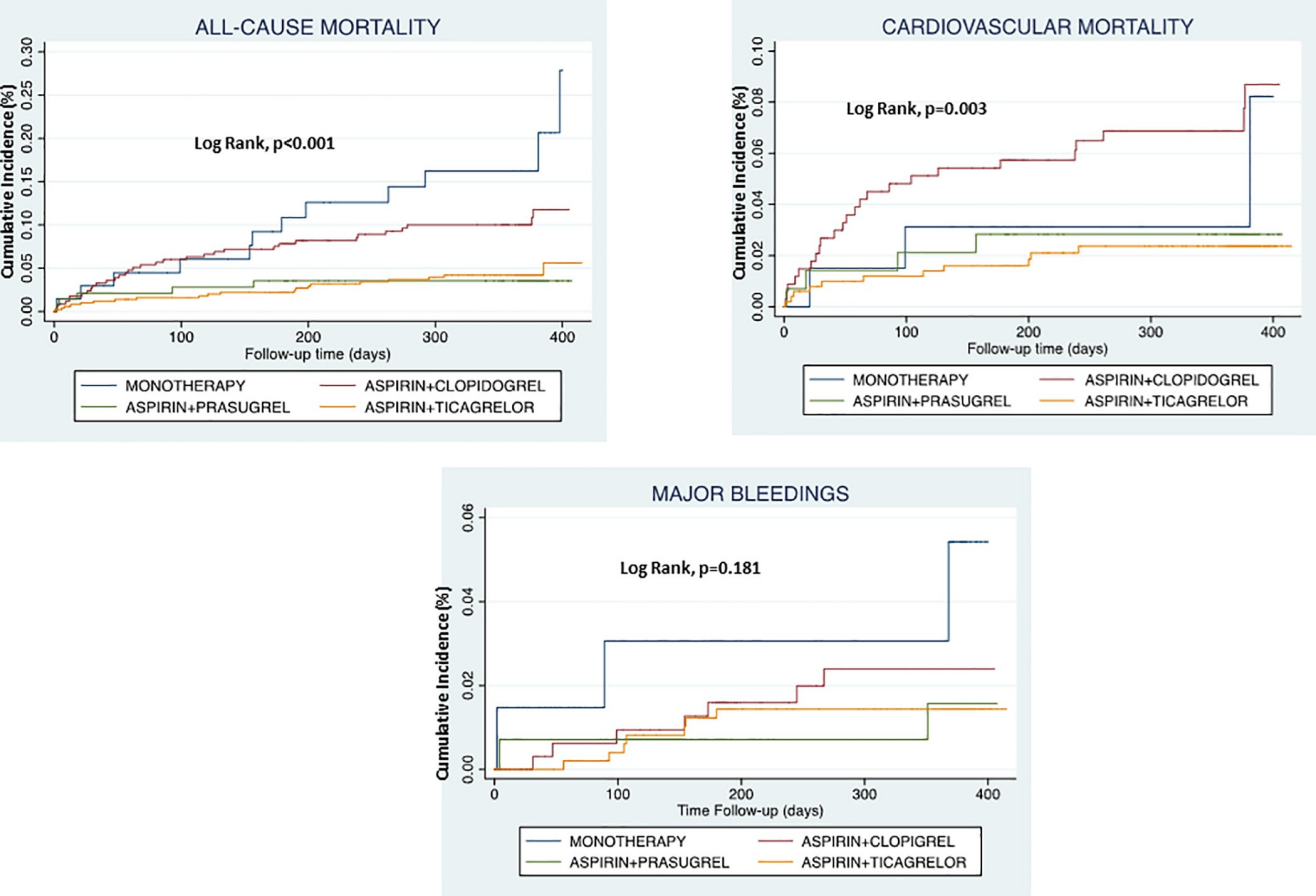
Kaplan Meier survival curves for outcomes.

The event-free survival curves for all-cause death, cardiovascular death and hemorrhagic complications according to the 4 groups of antiplatelet treatment are shown in Fig 1.

As the number of ACS patients treated with a monotherapy is low, we performed the Cox regression analyses only in patients treated with a dual antiplatelet treatment (i.e. aspirin+clopidogrel, aspirin+prasugrel and aspirin+ticagrelor).

As shown in Tables 5, 6 and 7, at the multivariable Cox regression analysis the duration of dual antiplatelet treatments was independently and significantly associated with all-cause mortality, cardiovascular mortality and major bleedings after adjustment of several potential confounders, including the type of dual antiplatelet treatment. In particular, a 12 month-duration of antiplatelet therapies was more effective in protecting ACS patients from all-cause, cardiovascular mortality and major bleeding complications with respect to a duration from 1 to 6 months.

**Table 5:** Univariable and multivariable predictors of All-cause mortality.

	Unadjusted HR (95%CI)	P value	Adjusted* HR (95% CI)	P value
**Age (years), mean (SD)**	1.07 (1.05–1.10)	<0.001	1.04 (1.01–1.08)	0.011
**Age ≥ 75 y**	3.96 (2.44–6.46)	<0.001	-	-
**Gender, males**	1.30 (0.79–2.14)	0.294	-	-
**BMI ≥ 30 Kg/m2**	1.07 (0.61–1.89)	0.814	-	-
**LVEF<40%**	5.26 (3.29–8.38)	<0.001	2.18(1.15–4.12)	0.016
**Hypertension**	0.83 (0.50–1.37)	0.464	-	-
**Hypercholesterolemia**	0.70 (0.44–1.12)	0.137	-	-
**Smoking habit**	0.59 (0.36–0.96)	0.033	1.08 (0.58–2.03)	0.802
**Diabetes**	1.84 (1.15–2.96)	0.012	1.21 (0.63–2.31)	0.561
**Family history for CAD**	0.57 (0.32–1.03)	0.620	-	-
**Previous Myocardial infarction**	1.40 (0.82–2.30)	0.214	-	-
**Previous PCI, n (%)**	1.60 (0.95–2.68)	0.076	1.40 (0.71–2.65)	0.306
**Non Valvular Atrial Fibrillation**	2.19 (1.18–4.09)	0.014	1.56 (0.67–3.63)	0.303
**Valvular Atrial Fibrillation**	6.10 (2.22–16.76)	<0.001	1.96 (0.40–9.64)	0.410
**Peripheral Artery Disease**	3.06 (1.68–5.59)	<0.001	1.97 (0.90–4.28)	0.089
**Chronic Kidney Disease**	4.80 (2.63–8.77)	<0.001	1.12 (0.50–2.54)	0.777
**Cerebrovascular disease**	2.41 (0.97–5.98)	0.059	1.29 (0.41–4.16)	0.665
**Previous Major Bleeding**	1.60 (0.39–6.53)	0.513	-	-
**Unstable Angina**	1	-	-	-
**NSTEMI**	2.78 (0.97–7.92)	0.056	3.54 (0.7–17.95)	0.126
**STEMI**	2.46 (0.88–6.90)	0.088	3.97 (0.77–20.39)	0.099
**Proton Pump Inhibitors**	0.93 (0.23–3.78)	0.914	-	-
**Statins**	0.61 (0.22–1.68)	0.343	-	-
**Beta-Blockers**	0.46 (0.29–0.73)	0.001	0.74 (0.41–1.33)	0.313
**Diuretics**	2.93 (1.84–4.69)	<0.001	1.09 (0.60–1.99)	0.770
**ACE inhibitors/ARBS**	0.40 (0.25–0.64)	<0.001	0.80 (0.42–1.51)	0.485
**Nitrates**	1.77 (0.95–3.31)	0.072	0.87 (0.38–1.99)	0.743
**Vitamin K Antagonists**	1.64 (0.81–3.31)	0.168	-	-
**Antiplatelet Therapies**				
**Aspirin+Clopidogrel**	1			
**Aspirin+Prasugrel**	0.33 (0.12–0.83)	0.019	2.45 (0.78–7.67)	0.123
**Aspirin+Ticagrelor**	0.38 (0.22–0.68)	0.001	1.36 (0.68–2.71)	0.382
**Duration of Antiplatelet Therapies (days)**	0.99 (0.988–0.992)	<0.001	-	-
**Duration of Antiplatelet Therapies (for each SD increase)**	0.38 (0.22–0.67)	0.001	0.20 (0.14–0.28)	<0.001

**Table 6:** Univariable and multivariable predictors of cardiovascular mortality.

	Unadjusted HR (95%CI)	P value	Adjusted* HR (95% CI)	P value
**Age (years), mean (SD)**	1.07 (1.04–1.11)	<0.001	1.05 (1.01–1.09)	0.020
**Age ≥ 75 y**	3.49 (1.87–6.52)	<0.001	-	-
**Gender, males**	1.50 (0.80–2.82)	0.210	-	-
**BMI ≥ 30 Kg/m2**	1.09 (0.52–2.28)	0.821	-	-
**LVEF<40%**	6.00 (3.28–11.0)	<0.001	2.81 (1.26–6.23)	0.011
**Hypertension**	1.06 (0.53–2.12)	0.876	-	-
**Hypercholesterolemia**	0.75 (0.41–1.37)	0.394	-	-
**Smoking habit**	0.51 (0.27–0.97)	0.041	1.26 (0.56–2.86)	0.577
**Diabetes**	2.31 (1.26–4.25)	0.007	1.69 (0.74–3.85)	0.212
**Family history for CAD**	0.73 (0.36–1.49)	0.394	-	-
**Previous Myocardial infarction**	2.33 (1.23–4.38)	0.009	2.35 (0.87–6.43)	0.093
**Previous PCI, n (%)**	2.28 (1.21–4.28)	0.011	0.89 (0.33–2.44)	0.825
**Non Valvular Atrial Fibrillation**	2.60 (1.20–5.62)	0.015	2.00 (0.73–5.40)	0.177
**Valvular Atrial Fibrillation**	7.74 (2.39–25.06)	<0.001	4.29 (0.78–3.54)	0.094
**Peripheral Artery Disease**	3.21 (1.49–6.95)	0.003	1.23 (0.45–3.36)	0.685
**Chronic Kidney Disease**	6.53 (3.21–13.29)	<0.001	1.42 (0.55–3.71)	0.471
**Cerebrovascular disease**	2.50 (0.77–8.08)	0.127	-	-
**Previous Major Bleeding**	1.32 (0.18–9.60)	0.784	-	-
**Unstable Angina**	1	-	-	-
**NSTEMI**	2.12 (0.62–7.23)	0.232	4.67 (1.54–14.13)	0.006
**STEMI**	1.84 (0.55–6.14)	0.323	7.62 (2.49–23.34)	<0.001
**Proton Pump Inhibitors**	1.07 (0.15–7.78)	0.947	-	-
**Statins**	1.47 (0.20–10.71)	0.702	-	-
**Beta-Blockers**	0.32 (0.17–0.59)	<0.001	0.66 (0.31–1.41)	0.283
**Diuretics**	1.93 (1.04–3.55)	0.036	0.50 (0.23–1.09)	0.083
**ACE inhibitors/ARBS**	0.32 (0.17–0.63))	<0.001	0.62 (0.28–1.38)	0.241
**Nitrates**	1.79 (0.79–4.03)	0.161	-	-
**Vitamin K Antagonists**	1.95 (0.82–4.63)	0.130	-	-
**Antiplatelet Therapies**				
**Aspirin+Clopidogrel**	1			
**Aspirin+Prasugrel**	0.37 (0.13–1.07)	0.067	3.89 (1.01–15.0)	0.048
**Aspirin+Ticagrelor**	0.30 (0.15–0.61)	0.001	1.07 (0.44–2.61)	0.874
**Duration of Antiplatelet Therapies (days)**	0.989 (0.986–0.991)	<0.001	-	-
**Duration of Antiplatelet Therapies (for each SD increase)**	0.23 (0.17–0.33)	<0.001	0.14 (0.09–0.23)	<0.001

**Table 7:** Univariable and multivariable predictors of major bleedings.

	Unadjusted HR (95%CI)	P value	Adjusted* HR (95% CI)	P value
**Age (years), mean (SD)**	1.02 (0.98–1.06)	0.303	-	-
**Age ≥ 75 y**	2.41 (0.98–5.93)	0.056	1.36 (0.44–4.24)	0.597
**Gender, males**	0.97 (0.35–2.69)	0.954	-	-
**BMI ≥ 30 Kg/m2**	0.47 (0.11–2.03)	0.310	-	-
**LVEF<40%**	3.61 (1.42–9.18)	0.007	2.71(0.90–8.20)	0.078
**Hypertension**	0.80 (0.30–2.10)	0.646	-	-
**Hypercholesterolemia**	0.99 (0.41–2.46)	0.999	-	-
**Smoking habit**	0.72 (0.29–1.78)	0.469	-	-
**Diabetes**	1.02 (0.37–2.83)	0.971	-	-
**Family history for CAD**	0.62 (0.21–1.86)	0.391	-	-
**Previous Myocardial infarction**	0.23 (0.03–1.71)	0.151	-	-
**Previous PCI, n (%)**	0.48 (0.11–2.08)	0.328	-	-
**Non Valvular Atrial Fibrillation**	3.80 (1.35–10.72)	0.011	2.89 (0.56–14.84)	0.203
**Valvular Atrial Fibrillation**	12.92 (2.98–56.0)	0.001	7.04 (0.69–71.63)	0.099
**Peripheral Artery Disease**	0.77 (0.10–5.82)	0.805	-	-
**Chronic Kidney Disease**	1.21 (0.16–9.08)	0.852	-	-
**Cerebrovascular disease**	1.74 (0.23–13.07)	0.591	-	-
**Previous Major Bleeding**	3.19 (0.43–23.91)	0.259	-	-
**Unstable Angina**	1	-	-	-
**NSTEMI**	0.79 (0.20–3.20)	0.789	-	-
**STEMI**	0.89 (0.24–3.25)	0.886	-	-
**Proton Pump Inhibitors**	0.50 (0.07–3.80)	0.505	-	-
**Statins**	0.65 (0.09–4.85)	0.671	-	-
**Beta-Blockers**	0.57 (0.23–1.42)	0.229	-	-
**Diuretics**	2.82 (1.14–7.00)	0.025	1.70 (0.58–5.00)	0.333
**ACE inhibitors/ARBS**	0.58 (0.24–1.42)	0.232	-	-
**Nitrates**	0.99 (0.23–4.36)	0.995	-	-
**Vitamin K Antagonists**	2.95 (0.96–9.05)	0.059	0.51 (0.08–3.15)	0.472
**Antiplatelet Therapies**				
**Aspirin+Clopidogrel**	1			
**Aspirin+Prasugrel**	0.63 (0.13–3.02)	0.562	2.16 (0.36–12.99)	0.400
**Aspirin+Ticagrelor**	0.64 (0.22–1.82)	0.402	1.44 (0.42–4.95)	0.558
**Duration of Antiplatelet Therapies (days)**	0.994 (0.991–0.997)	<0.001	-	-
**Duration of Antiplatelet Therapies (for each SD increase)**	0.45 (0.31–0.65)	0.001	0.39 (0.24–0.65)	<0.001

When the models were corrected for the competing risks, we obatained similar results.

## Discussion

The analysis of the register has documented:

1. the most prevalent P2Y12 inhibitor used in association with aspirin is ticagrelor independenlty of concomitant risk factors and type of revascularization;
2. both ticagrelor and prasugrel are associated with a statistically significant reduced total and cardiovascular mortality at univariate analysis. Kaplan Meier curves are similar for both groups and significantly diverge with respect to aspirin alone or aspirin plus clopidogrel;
3. both prasugrel and ticagrelor do not show a significant increased incidence of major and minor bleedings with respect to clopidogrel;

As regards as the clinical characteristics of ACS, we have registered a slight higher incidence of STEMI with respect to NSTEMI. Investigators have defined a priori how to enroll patients: for example, all patients in the first or second week of the month, in order to eliminate possible selection bias. For this reason, this datum mirrors the clinical real world.

Accordingly to literature data [7], more than eighty percent of patients received a percutaneous revascularization: among these, more than 90% received drug eluting stent implantation. 2.3% of patients underwent surgical revascularization; and 14.5% was treated by medical therapy alone.

At hospital discharge, almost the totality of patients was prescribed proton pump inhibitors [more prevalent omeprazole (44.9%) and pantoprazole (36.3%)] and statin [more prevalent atorvastatin (93%)].

Despite the presence in the literature of data regarding the negative association between clopidogrel and PPI and ticagrelor and PPI [8,9, 10, 11] almost the totality of patients was prescribed PPIs.

This datum indicates that from a clinical point of view, clinicians do not worry about the possible ischemic risk associated with the combination between PPIs and dual antiplatelet treatment, whereas the perceived risk of gastrointestinal bleedings prevails.

The most prevalent prescription of atorvastatin is in line with literature data and guidelines [12, 13].

One hundred and two patients (9.7%) received, at the time of discharge, an oral anticoagulant therapy. The most prescribed oral anticoagulant was warfarin (83/102; 81.4%) and in 13.7% the oral direct anticoagulant dabigratan.

In this registry we have documented that the ‘old’ clopidogrel remains the second most prescribed antiplatelet drug with a significant prevalence of about 32%. The association aspirin plus clopidogrel was more prevalent in patients with advanced age, with lower BMI, lower hemoglobin levels, creatinine clearance, and with more comorbidities (previous peripheral artery disease; previous TIA/stroke; previous major bleeding) with respect to patients treated with aspirin plus ticagrelor or aspirin plus prasugrel.

At the opposite, aspirin plus prasugrel was used in younger patients; with higher BMI and GFR; with lower prevalence of history of PAD or previous MI. No patient in the group aspirin plus prasugrel had a history of major or minor bleeding. In diabetics, aspirin plus prasugrel was the less prescribed association, according to recent published data [14].

These data document that the choice of aspirin and clopidogrel is prevalent in the populations with more frailty: older patients, with a decreased renal function and lower levels of hemoglobin. That is, from a clinical point of view, the choice of a less potent antiplatelet in patients perceived at higher risk of bleeding. On the other hand, this association was more used also in patients with higher comorbidities, such as previous peripheral artery disease or myocardial infarction: this choice appears to be less appropriate.

Total and cardiovascular mortality is significantly higher in aspirin plus clopidogrel with respect to the other two associations.

At the univariate Cox regression analyses aspirin plus ticagrelor and aspirin plus prasugrel were both associated with a significantly lower risk for total and cardiovascular mortality. At the multivariate analyses, independent predictors of cardiovascular death were: age, reduced ejection fraction (lower than 40%), previous stroke, renal insufficiency and the duration of dual antiplatelet treatment.

Kaplan Meier curves significantly diverge and show a higher and early all-cause mortality (within 2 months) in patients treated only with aspirin or with aspirin plus clopidogrel: the early timing of the event might underlie the frailty of these patients in which the clinicians decided to use only one antiplatelet drug or the less potent combination of antiplatelet drugs, apparently in disagreement with current guidelines.

The curve of patients treated by aspirin plus clopidogrel diverges in a clear way from those of aspirin plus ticagrelor and aspirin plus prasugrel and shows later events, between 8 and 10 months from diagnosis.

As regards as ischemic events at follow-up, the half was registered in aspirin alone and aspirin plus clopidogrel groups. No cerebral ischemic event in patients treated with aspirin plus prasugrel or aspirin plus ticagrelor was registered. Even recurrent myocardial infarction was documented with a significant more prevalence in aspirin alone group and aspirin plus clopidogrel.

Moving to the analysis of safety end-points, we have evaluated these events using three different scores: GUSTO [5], TIMI [4] and ISTH [6]. Using all scores, data show a paradoxical higher bleeding risk, major and minor, in patients treated with aspirin alone, again according to the frailty of these patients which determined the prescription of a single antiplatelet.

On the other hand, in the presence of a dual antiplatelet therapy, no significant differences, in the incidence of major bleeding, were documented between the association aspirin plus ticagrelor/prasugrel or aspirin plus clopidogrel. These data are clinically relevant as suggest how in the real world the incidence of bleedings in patients treated with the more potent P2Y12 inhibitors do not seem to be higher than with the administration of clopidogrel. This might reflect the use of these drugs in a populations at lower bleeding risk (younger patients, with higher BMI, with higher GFR). Reduced ejection fraction and previous stroke are independent predictors of bleedings in this cohort.

The duration of dual antiplatelet therapy was confirmed [15, 16] to be an independent and strong predictor of both thrombotic and bleeding events.

Limitations of the study are: 1) there are considerable baseline differences among the antiplatelet groups which potentially affect the results derived from multiple statistical adjustments; 2) the diagnosis of myocardial infarction is based only on any biomarker increase, ecg or echo abnormalities, excluding myocardial ischemia which may result in small biomarker increase in the absence of myocardial necrosis.

In conclusion, the START antiplatelet registry document that aspirin plus ticagrelor is the first choice of antiplatelet, but the ‘old’ clopidogrel is yet prescribed in a significant proportion of ACS patients. In the real world, the datum of a reduced mortality associated with the use of ticagrelor was confirmed and was present also for prasugrel. On the other hand, no significant higher prevalence of major bleeding events in patients treated with the more potent P2Y12 antiplatelets was documented.
